# A Treatise on Diseases of the Rectum, Anus, and Sigmoid Flexure

**Published:** 1903-11

**Authors:** 


A Treatise on Diseases of the Rectum, Anus, and Sigmoid Flexure.—By Jos. M. Mathews, M. D., LL. D., Professor of Surgery, and Clinical Lecturer on Diseases of the Rectum, Hospital College of Medicine. With six chromo-lithographs and numerous illustrations. Third edition, revised. Price, $5.00. Published by D. Appleton & Co., New York.

This, the third edition, contains much new material. The article on cancer has been rewritten for the present volume, and it is one of the most thorough and scientific articles on the subject that we have been able to find anywhere. A good deal of space is devoted to the very full description of several operations devised by the author. The technique of these in the main appeals to reason as being very satisfactory. An interesting chapter is added on rectal valves.

The sale of the former editions insures the reception of this one.

